# EGA Protects Mammalian Cells from *Clostridium difficile* CDT, *Clostridium perfringens* Iota Toxin and *Clostridium botulinum* C2 Toxin

**DOI:** 10.3390/toxins8040101

**Published:** 2016-04-01

**Authors:** Leonie Schnell, Ann-Katrin Mittler, Mirko Sadi, Michel R. Popoff, Carsten Schwan, Klaus Aktories, Andrea Mattarei, Domenico Azarnia Tehran, Cesare Montecucco, Holger Barth

**Affiliations:** 1 Institute of Pharmacology and Toxicology, University of Ulm Medical Center, Albert-Einstein-Allee 11, 89081 Ulm, Germany; leonie.schnell@uni-ulm.de (L.S.); ann-katrin.mittler@uni-ulm.de (A.-K.M.); mirko.sadi@uni-ulm.de (M.S.); 2 Department of Anaerobic Bacteria, Pasteur Institute, 75015 Paris, France; michel-robert.popoff@pasteur.fr; 3 Institute of Experimental and Clinical Pharmacology and Toxicology, University of Freiburg, 79104 Freiburg, Germany; carsten.schwan@pharmakol.uni-freiburg.de (C.S.); klaus.aktories@pharmakol.uni-freiburg.de (K.A.); 4 Department of Chemical Sciences, University of Padova, 35121 Padova, Italy; andrea.mattarei@unipd.it; 5 Department of Biomedical Sciences, University of Padova, 35121 Padova, Italy; doazte@gmail.com (D.A.T.); cesare.montecucco@gmail.com (C.M.)

**Keywords:** *Clostridium difficile* CDT, *Clostridium perfringens* iota toxin, *Clostridium botulinum* C2 toxin, binary toxin, EGA

## Abstract

The pathogenic bacteria *Clostridium difficile*, *Clostridium perfringens* and *Clostridium botulinum* produce the binary actin ADP-ribosylating toxins CDT, iota and C2, respectively. These toxins are composed of a transport component (B) and a separate enzyme component (A). When both components assemble on the surface of mammalian target cells, the B components mediate the entry of the A components via endosomes into the cytosol. Here, the A components ADP-ribosylate G-actin, resulting in depolymerization of F-actin, cell-rounding and eventually death. In the present study, we demonstrate that 4-bromobenzaldehyde *N*-(2,6-dimethylphenyl)semicarbazone (EGA), a compound that protects cells from multiple toxins and viruses, also protects different mammalian epithelial cells from all three binary actin ADP-ribosylating toxins. In contrast, EGA did not inhibit the intoxication of cells with *Clostridium difficile* toxins A and B, indicating a possible different entry route for this toxin. EGA does not affect either the binding of the C2 toxin to the cells surface or the enzyme activity of the A components of CDT, iota and C2, suggesting that this compound interferes with cellular uptake of the toxins. Moreover, for C2 toxin, we demonstrated that EGA inhibits the pH-dependent transport of the A component across cell membranes. EGA is not cytotoxic, and therefore, we propose it as a lead compound for the development of novel pharmacological inhibitors against clostridial binary actin ADP-ribosylating toxins.

## 1. Introduction

The pathogenic clostridia *Clostridium* (*C.*) *difficile*, *C. perfringens* and *C. botulinum* produce the binary protein toxins CDT [1,2,3,4], iota [5,6,7,8] and C2 [9,10,11], respectively, which enter mammalian cells and directly modify the actin cytoskeleton, which results in cell-rounding and, finally, apoptotic cell death. These toxins are composed of two separate proteins, which must form complexes on mammalian target cells to exhibit their cytotoxic effects [12,13]. The proteolytically-activated binding/translocation (B) components of these toxins form ring-shaped heptamers (B_7_), which bind to their cell surface receptors [14,15,16,17]. CDTb and Ib, the B components of CDT and iota toxin, respectively, bind to a protein receptor on the cell surface, namely the lipolysis-stimulated receptor (LSR) [18,19,20]. In addition to LSR, the integrin CD44 is also involved in the binding of CDT and Ib to cells and in the internalization of both toxins [21]. In contrast, the B component of C2 toxin (C2IIa) binds to the asparagine-linked complex and hybrid carbohydrate structures present on all eukaryotic cell types [22].

The A components of the binary toxins bind to their respective cell-bound B components, and the AB_7_-toxin complexes are internalized by receptor-mediated endocytosis [23,24,25,26]. Within the acidic lumen of endosomes, the B_7_ oligomer changes conformation and inserts into the endosomal membranes, forming trans-membrane channels, which allow the translocation of the unfolded A components from the endosomal lumen into the cytosol [14,15,23,24,25,26,27,28,29,30,31]. Besides the pores and the acidic conditions, particular host cell chaperones are crucial for the translocation of enzymatically-active A components into the host cell cytosol [32,33,34,35,36,37]. Once in the cytoplasm, the A components mono-ADP-ribosylate G-actin at arginine-177 [9,38,39,40,41,42,43,44,45], and this covalent actin modification inhibits actin polymerization, resulting in a variety of direct and indirect adverse cellular effects, including cell-rounding, loss of barrier functions in the polarized epithelial layers, cell death and enhanced adherence of clostridia to gut epithelial cells [46,47,48,49,50,51,52].

The clostridial binary toxins are potent enterotoxins and cause severe diseases in humans and animals. From a medical point of view, CDT contributes to the severe forms of *C. difficile* infections (CDI). *C. difficile* causes severe enteric diseases in hospitalized patients treated with broad-spectrum antibiotics. The spectrum of CDI ranges from diarrhea to severe, potentially life-threatening pseudomembranous colitis due to the disturbed gut flora, which allows spore germination and the growth of *C. difficile* [53]. Protein toxins, including the large toxins A (TcdA, 308 kDa) and B (TcdB, 270 kDa), which mono-glucosylate the GTPases Rho, Rac and Cdc42 in the cytosol of mammalian cells, are the causative agents of *C. difficile*-associated diseases. These toxins inhibit signal transduction via these Rho GTPases, resulting in the destruction of the actin cytoskeleton, cell-rounding and loss of intestinal wall integrity [54,55,56]. However, about 6%–35% of the *C. difficile* strains produce CDT in addition to toxins A and B, and this most likely contributes to their hypervirulence and the increased morbidity/mortality of patients infected with these strains [57,58,59,60,61].

Because such hypervirulent strains are resistant to broad-spectrum antibiotics used to treat other bacterial infections and allow overgrowth of *C. difficile*, the development of pharmacological inhibitors, acting directly on the toxins and protecting cells from intoxication, is an important goal. By performing high-throughput screening from a library of small molecules to find novel inhibitors against the anthrax lethal toxin [62], Bradley and co-workers identified the compound 4-bromobenzaldehyde *N*-(2,6-dimethylphenyl)semicarbazone (EGA) as a potent inhibitor against multiple bacterial toxins and viruses, which require trafficking through acidic vesicles to enter the host cell cytosol [63]. In particular, they found that EGA inhibits the cytotoxic effects that are caused by the anthrax lethal factor in the cytosol of host cells, suggesting that it might interfere with toxin trafficking between early and late endosomes; however, the molecular mechanism underlying the inhibitory effect of EGA is not known [63]. More recently, an optimized synthesis procedure for EGA was developed, and it was found that this compound prevents the neuroparalytic effects of different botulinum neurotoxin (BoNT) serotypes, most likely by interfering with toxin trafficking [64].

Prompted by these findings, we investigated the effect of EGA on the binary clostridial actin ADP-ribosylating toxins CDT, iota and C2, which also require trafficking through acidified endosomal vesicles in order to identify a novel pharmacological inhibitor against this toxin family.

## 2. Results and Discussion

**Figure 1:**
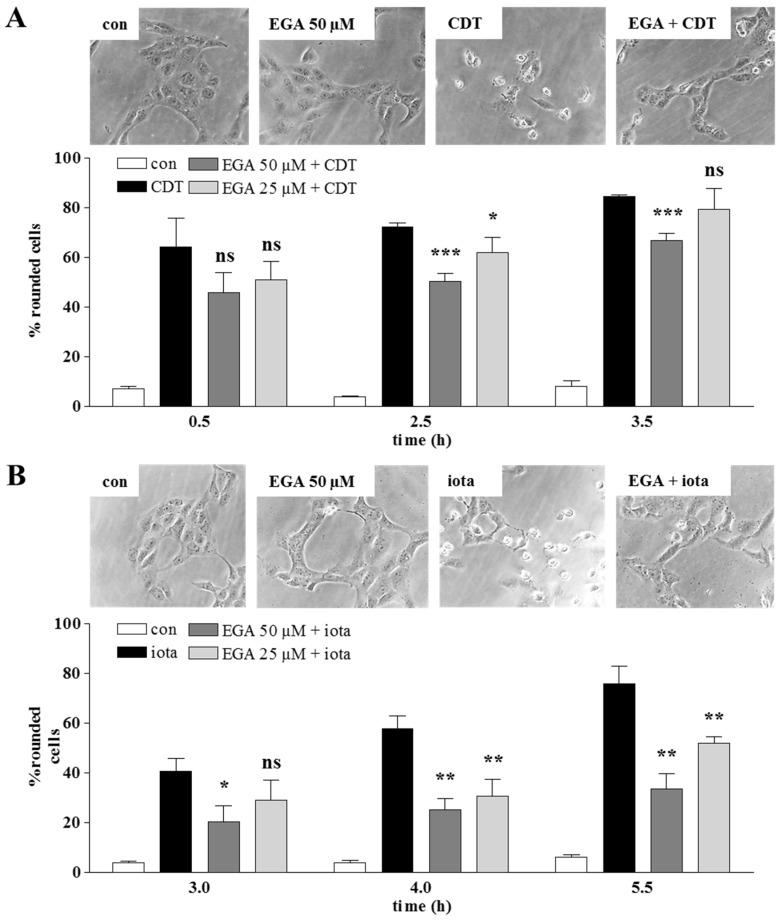

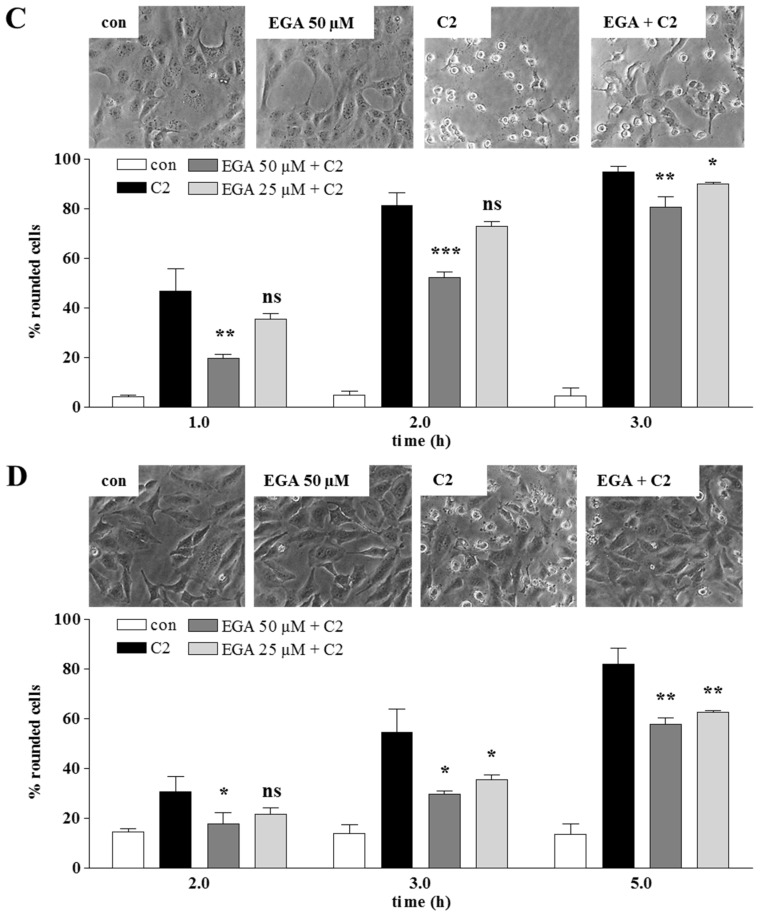
Pre-treatment with EGA protects cells from intoxication with the binary clostridial toxins CDT, iota and C2. Vero or HeLa (for C2 in addition to Vero) cells were pre-incubated for 1 h at 37 °C with 25 and 50 µM EGA or the solvent DMSO. Thereupon, CDT (CDTa: 30 ng/mL; CDTb: 60 ng/mL) (**A**), iota (Ia: 25 ng/mL; Ib: 50 ng/mL) (**B**) or C2 (C2I: 50 ng/mL; C2IIa: 100 ng/mL) ((**C**): Vero; (**D**): HeLa) was added, and cells were further incubated in the presence of the toxin. For the control (con), cells were left untreated or incubated with the respective toxin alone in the absence of EGA. At the indicated time points, pictures were taken with a Zeiss Axiovert microscope, and the percentage of rounded cells was determined for quantitative analysis. Representative pictures after 2.5 h (CDT), 4 h (iota), 2 h (C2 on Vero cells) and 3 h (C2 on HeLa cells) are shown. Values are given as the mean ± SD (*n* = 3); significance was tested between cells treated with the respective toxin in the absence (black bars) and presence of EGA (grey bars) using Student’s *t*-test (n.s., not significant; * *p* < 0.05, ** *p* < 0.01, *** *p* < 0.001).

EGA protects cells from intoxication with the binary actin ADP-ribosylating toxins CDT, iota and C2. Vero cells were treated with each of the binary toxins in the presence or absence of EGA, and the intoxication process was monitored in terms of cell-rounding, which is a well-established, highly specific and sensitive endpoint to detect the uptake of the A components of these toxins into the host cell cytosol. Vero cells express the receptor for CDT and iota toxin and were therefore used as a model cell line in this study. As shown in Figure 1, these cells treated with the enzyme plus the binding components of the toxins rounded up due to the ADP-ribosylation of G-actin in the cytosol. Cells that were pre-treated with EGA showed less cell-rounding, indicating that this molecule interferes with the mode of action of CDT, iota toxin and C2 toxin (Figure 1A–C). For C2 toxin, the same experiment was performed using also HeLa cells, and we obtained almost an identical level of protection (Figure 1D). EGA delayed the intoxication with C2 toxin in a concentration-dependent manner, as tested for EGA concentrations from 6–100 µM (data not shown). An increased period of EGA pretreatment did not result in an enhanced protection of cells from C2 toxin (data not shown).

**Figure 2:**
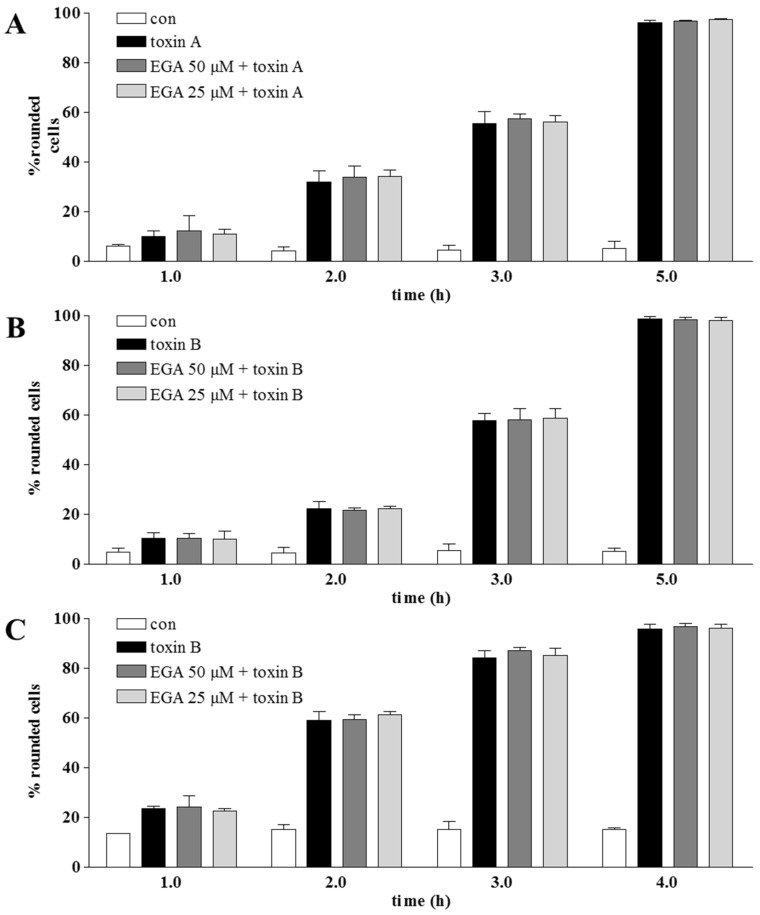
Pre-treatment with EGA has no effect on the intoxication with *C. difficile* toxins A and B. Vero or HeLa (for toxin B in addition to Vero) cells were pre-incubated for 1 h at 37 °C with 25 and 50 µM EGA or the solvent DMSO. Thereupon, toxin A (7 ng/mL) (**A**) or toxin B (1 ng/mL) ((**B**): Vero; (**C**): HeLa) was added, and cells were further incubated in the presence of the toxin. For the control, cells were left untreated or incubated with the respective toxin alone in the absence of EGA. At the indicated time points, pictures were taken, and the percentage of rounded cells was determined for quantitative analysis. Values are gives as the mean ± SD (*n* = 3).

In contrast, EGA did not inhibit the intoxication of cells with the large Rho-glucosylating *C. difficile* toxins A (TcdA) and B (TcdB) (Figure 2). Like the binary actin ADP-ribosylating toxins, these toxins are internalized by receptor-mediated endocytosis and deliver their enzymatically-active subunits from acidic endosomal vesicles into the host cell cytosol [56]. Therefore, this result suggests that EGA might not interfere with the cellular process in general, but more specifically with regard to the individual trafficking mechanisms, which are exploited by the respective bacterial toxin. Thus, *C. difficile* toxins A and B might exploit different cellular trafficking mechanisms compared to CDT, iota and C2.

**Figure 3:**
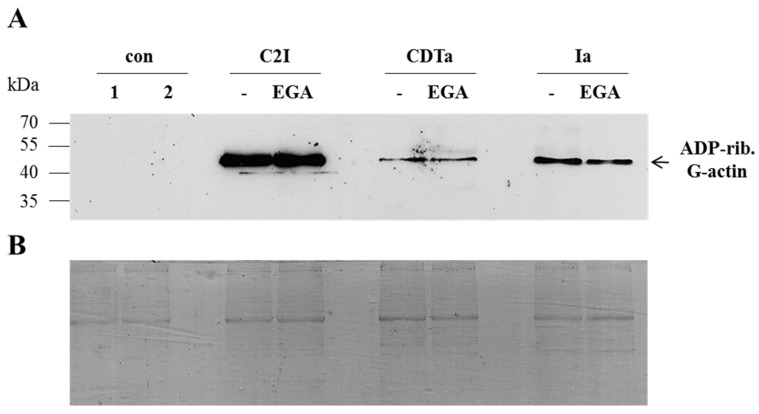
*In vitro*, treatment with EGA has no effect on the enzyme activity of CDTa, Ia or C2I. (**A**) C2I, CDTa or Ia (100 ng each) was pre-incubated for 10 min at 37 °C with 50 µM EGA or left untreated for the control. Thereafter, 20 µg of Vero lysate protein and 10 µM biotin-labelled NAD^+^ were added, and the samples were incubated at 37 °C for 10 min. Then, samples were subjected to SDS-PAGE and blotted, and biotinylated (*i.e.*, ADP-ribosylated, indicated as ADP-rib.) G-actin was detected. For the control, a sample of only Vero lysate (1) and one of Vero lysate with biotin-labelled NAD^+^ (2) were additionally analyzed. (**B**) Comparable amounts of total protein were confirmed by Coomassie staining of the proteins in the SDS-gel after the blotting process.

Based on these findings, we investigated the mechanism underlying the inhibitory effect of EGA towards the binary actin ADP-ribosylating toxins in more detail. First, it was tested whether EGA inhibits the enzyme activity of the A components. To this end, cell lysate was incubated with each of the enzyme components together with biotin-labelled NAD^+^ as the co-substrate in the absence and presence of EGA, and the ADP-ribosylated, *i.e.*, biotin-labelled, actin was analyzed by Western blotting (Figure 3). Taken together, there were comparable amounts of ADP-ribosylated actin, independent of whether EGA was present or not.

This result indicates that EGA did not inhibit the enzyme activity of CDTa, Ia or C2I *in vitro*. However, it cannot be excluded that this compound has inhibitory effects on the enzyme activity of the toxins *in vivo*.

**Figure 4:**
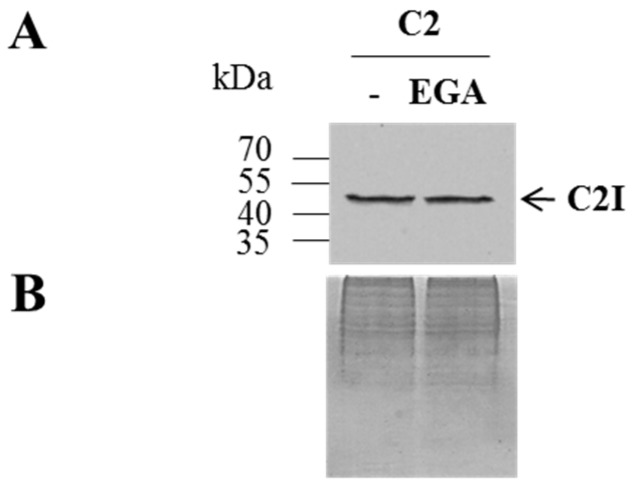
Treatment with EGA has no effect on the binding of C2 toxin to cells. (**A**) HeLa cells were pre-treated in serum-free medium with 50 µM EGA for 1 h at 37 °C or left untreated for the control. Afterwards, cells were kept on ice. After 10 min, C2 (C2I: 1 µg/mL; C2IIa: 2 µg/mL) was added followed by further incubation at 4 °C for 30 min to enable receptor binding of C2. Thereafter, unbound toxin was removed by three washing steps with ice-cold PBS, and cells were scraped off in five-fold SDS sample buffer containing 10% DTT; proteins were separated by SDS-PAGE and blotted, and bound C2I (*i.e.*, bound C2IIa) was detected using a specific antibody against C2I and peroxidase-coupled anti-rabbit antibody by the enhanced chemiluminescence (ECL) system. As the control of equal protein loading, the SDS-gel was stained with Coomassie (**B**).

Next, it was investigated whether EGA might interfere with the uptake of the A components into the host cell cytosol. C2 toxin was used to prove this hypothesis, because C2 represents the prototype of the family of binary actin ADP-ribosylating toxins. EGA did not inhibit the binding of C2 toxin to its receptor on the cell surface, as analyzed by Western blotting of cell-bound toxin (Figure 4).

**Figure 5:**
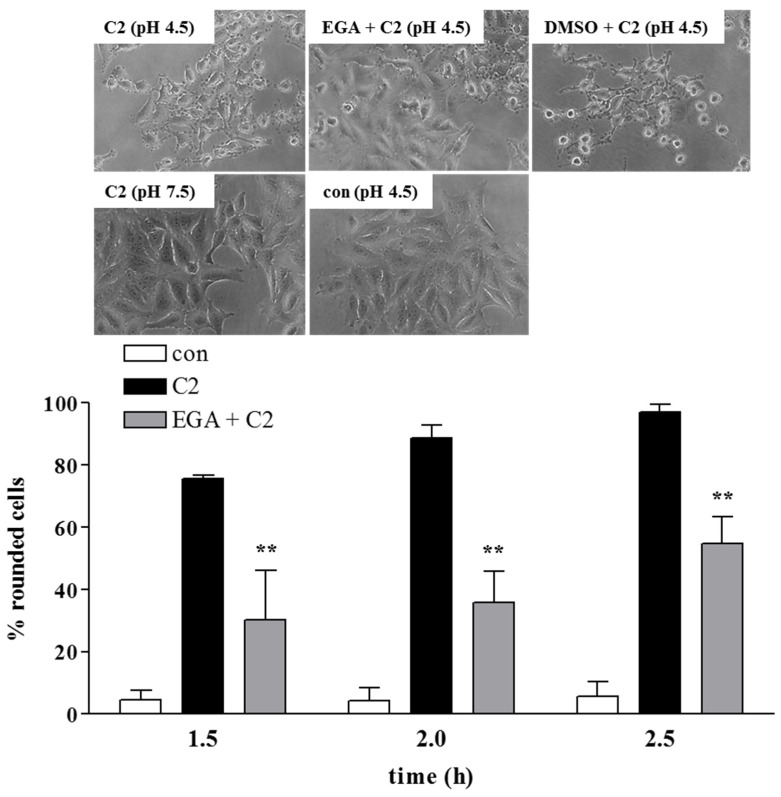
Pre-treatment with EGA inhibits the pH-dependent membrane transport of the enzyme component C2I of the binary C2 toxin in living cells. HeLa cells were pre-incubated in serum-free medium for 1 h at 37 °C with 100 nM bafilomycin (Baf) A1 plus 50 µM EGA or the solvent DMSO. Thereafter, cells were kept on ice for 10 min. Following this, C2 toxin (C2I: 75 ng/mL; C2IIa: 150 ng/mL) was added, and cells were incubated for 20 min at 4 °C to enable receptor binding. For the control, the cells were further incubated at pH 4.5 or pH 7.5 for 10 min at 37 °C. EGA was not added to the medium during this step. Thereupon, cells were incubated in neutral medium (pH 7.5) containing serum, Baf A1 ± EGA at 37 °C. After 1.5, 2 and 2.5 h, pictures were taken, and for quantitative analysis, the percentage of rounded cells was determined. Representative pictures after 2 h are shown. Values are given as the mean ± SD (*n* = 3); significance was tested between C2 and EGA + C2 by using Student’s *t*-test (** *p* < 0.01).

Finally, it was tested whether EGA-treatment of cells interferes with the pH-dependent transport of C2I across cell membranes into the cytosol. During the uptake of C2 toxin into cells, C2I translocates through pores that are formed by C2IIa in endosomal membranes from the lumen of acidified endosomal vesicles across endosomal membranes into the cytosol. This step can be investigated in a direct manner and independent from the other steps of toxin internalization by performing a well-established assay [14], which mimics the conditions in the lumen of acidified endosomal vesicles on the surface of living cells. In brief, cells were incubated at 4 °C with C2IIa plus C2I to enable the binding of both components of C2 toxin to the cells. Then, cells with bound C2 toxin were exposed to a short acidic pulse to trigger the insertion of the cell-bound C2IIa heptamers into the cytoplasmic membranes, where C2IIa forms translocation pores and C2I directly translocates through these pores across the membrane into the cytosol. Importantly, the normal uptake of C2 toxin via acidic endosomal vesicles was blocked by the treatment of the cells with bafilomycin A1, an inhibitor of v-ATPase. In the cytosol, the translocated C2I subsequently ADP-ribosylates G-actin and induces cell-rounding, which was monitored to determine the uptake of enzymatically-active C2I into the cytosol. In the presence of EGA, less cells rounded up, clearly indicating that EGA interferes with the pH-dependent membrane transport of C2I during the uptake of C2 toxin into cells (Figure 5). Since EGA was not added to the acidic medium during the pH pulse, it can be excluded that EGA elevates the pH value of the medium, thereby preventing pore formation and translocation. Moreover, intoxication of cells with C2 toxin was also reduced by lower concentrations of EGA (12.5 µM; not shown) that do not neutralize acidic organelles, as demonstrated earlier [63]. However, the precise mode of action of EGA in inhibiting the membrane transport of the toxin in mammalian cells is not known. So far, it could be speculated whether EGA blocks the formation of the C2IIa heptameric translocation channel, as described for PA63 oligomers [63], or interferes with the refolding of C2I in the host cell cytosol.

## 3. Experimental Section

### 3.1. Materials and Reagents

Cell culture media (MEM) and fetal calf serum were from Invitrogen (Karlsruhe, Germany). Cell culture materials were obtained from TPP (Trasadingen, Switzerland). The protein molecular weight marker Page Ruler prestained Protein ladder^®^ was from Thermo Fisher Scientific Inc. (Waltham, MA, USA). Complete^®^ protease inhibitor was purchased from Roche (Mannheim, Germany). Biotinylated NAD^+^ was supplied by R & D Systems GmbH (Wiesbaden-Nordenstadt, Germany). Bafilomycin (Baf) A1 was obtained from Calbiochem (Bad Soden, Germany), and 2-(4-bromobenzylidene)-*N*-(2,6-dimethylphenyl)hydrazinecarboxamide (EGA) was synthesized as described [64] and provided by Dr. Cesare Montecucco (Department of Biomedical Sciences, University of Padova, Padua, Italy). Streptavidin-peroxidase was purchased from Roche (Mannheim, Germany) and the enhanced chemiluminescence (ECL) system from Millipore (Schwalbach, Germany). The nitrocellulose blotting membrane was from Whatman^®^ (Dassel, Germany). CDTa and CDTb (from *C. difficile* strain 196) were expressed as recombinant His-tagged proteins in the *Bacillus megaterium* expression system and purified as described earlier [18]. Ia and Ib were purified as described earlier [44]. The recombinant C2I and C2IIa proteins were prepared and activated as described earlier [36,43]. Toxin A and toxin B were purified as described [54,55].

### 3.2. Cell Culture and Intoxication Assays

HeLa and African green monkey kidney (Vero) cells were cultivated at 37 °C and 5% CO_2_ in MEM medium, containing 10% heat-inactivated fetal calf serum, 1 mM sodium-pyruvate, 2 mM l-glutamine, 0.1 mM non-essential amino acids and 1% penicillin-streptomycin. Cells were trypsinized and reseeded three times per week for at most 30 times.

For intoxication experiments, cells were seeded in culture dishes and pre-incubated with EGA or the vehicle DMSO in serum-free medium for 1 h at 37 °C. Control cells were incubated without inhibitor. Subsequently, CDT, iota toxin, C2, toxin A or toxin B was added, and cells were further incubated at 37 °C with toxin plus inhibitor. After the given incubation periods, the cells were visualized by using a Zeiss Axiovert 40CFI microscope (Oberkochen, Germany) with a Jenoptik progress C10 CCD camera (Jena, Germany) to analyze the morphological changes caused by the toxins, and toxin-induced cell-rounding was taken as an indication of the intoxication process. Finally, cells were counted per picture (ImageJ software 1.45 (1.47v), NIH, Bethesda, MD, USA, 2013), and the amount of rounded cells was determined as a percent.

### 3.3. SDS-PAGE and Western Blotting

For immunoblot analysis, equal amounts of protein were subjected to SDS-PAGE according to the method of Laemmli [65]. Subsequently, the proteins were transferred to a nitrocellulose membrane (Whatman, Dassel, Germany). The membrane was blocked for 1 h at RT with 5% dry milk powder in PBS containing 0.1% Tween-20 (PBS-T) or alternatively overnight at 4 °C. For the detection of the biotin-labelled G-actin, the samples were probed with streptavidin-peroxidase. Bound C2I was detected by probing the membrane with a specific antibody against C2I [43] for 1 h followed by washing steps with PBS-T and an incubation with anti-rabbit antibody coupled to horseradish peroxidase (Santa-Cruz, Heidelberg, Germany). Finally, in each case, the membrane was washed and proteins were visualized using a chemiluminescence (ECL) system according to the manufacturer’s instructions. Equal amounts of protein were confirmed by Ponceau S staining of the membrane and Coomassie staining of the gel.

### 3.4. ADP-Ribosylation of Actin by C2I, CDTa and Ia in a Cell-Free System

C2I, CDTa or Ia (100 ng in each case) was pre-incubated for 10 min at 37 °C with EGA (50 µM) or left untreated for the control. Subsequently, Vero lysate (20 µg of protein) and biotin-labelled NAD^+^ (10 µM) were added followed by an incubation of the samples for 10 min at 37 °C. Thereupon, the proteins were subjected to SDS-PAGE and, after blotting onto a nitrocellulose membrane, biotin-labelled (*i.e.*, ADP-ribosylated) G-actin was detected using Western blotting.

### 3.5. Binding of C2 Toxin to Its Cell Surface Receptor

HeLa cells were pre-incubated in serum-free medium with EGA (50 µM) for 1 h at 37 °C or left untreated for the control. Afterwards, cells were kept at 4 °C. After 10 min, C2 was added followed by further incubation at 4 °C for 30 min to enable C2-binding to its cell surface receptor. Unbound C2 was subsequently removed by three washing steps with ice-cold PBS, and cells were scraped in 5-fold concentrated sample buffer containing 10% DTT [65]. Proteins were separated by SDS-PAGE and blotted, and bound C2I (*i.e.*, bound C2IIa) was detected using a specific antibody against C2I and peroxidase-coupled secondary antibody.

### 3.6. Toxin-Translocation across the Cytoplasmic Membrane of Living Cells

The pH-dependent translocation of C2 across endosomal membranes was experimentally mimicked on the cytoplasmic membranes of intact cells as described earlier [14]. Therefore, HeLa cells were pre-incubated with Baf A1 (100 nM) in order to block the regular toxin uptake. To test the influence of EGA on membrane translocation, cells were also pre-incubated with EGA (50 µM) or the vehicle DMSO for 1 h at 37 °C. Next, cells were kept on ice for 10 min followed by an incubation with C2 for 20 min at 4 °C that allowed binding of the toxin to the cell surface, and subsequently, cells were exposed to an acidic pulse (pH 4.5) for 10 min at 37 °C to trigger toxin-translocation across the surface membrane. For the control, additional wells with toxin-treated cells were treated with neutral medium (pH 7.5). Thereafter, all cells were further incubated at 37 °C in neutral medium containing FCS and Baf A1 (100 nM) ± EGA (50 µM). After the given incubation periods, C2-induced cell-rounding was monitored and documented by photography. Finally, cells were counted per picture, and the amount of rounded cells was determined as a percent.

### 3.7. Reproducibility of the Experiments

All experiments were performed independently at least two times, and the results from representative experiments are shown in the figures. The quantification was performed by calculating the values (*n* = 3) as the means ± standard deviation (SD) with the Prism4 Software (Version 4.0, GraphPad Software Inc., La Jolla, CA, USA, 2003).

## 4. Conclusions

We have found that the compound EGA delays the intoxication of different cultured mammalian cells with the binary clostridial toxins CDT, iota and C2, but not with the *C. difficile* toxins A and B. EGA had no effect on the enzyme activity of CDTa, Ia and C2I, but inhibited the pH-dependent membrane transport of C2I in living human cells. Interestingly, EGA was identified before as a potent inhibitor against the binary anthrax lethal toxin, which is related to the binary clostridial actin ADP-ribosylating toxins regarding the structure of the B component and the cellular uptake. Moreover, EGA protected cells and animals from botulinum neurotoxins, which are different from the binary toxins in their structure, but, just like the binary toxins, translocate their enzyme moiety from acidic intracellular compartments into the host cell cytosol. EGA does not protect cells from *C. difficile* toxins A and B, providing a formal proof that these toxins have different mechanisms to deliver their enzymatic active subunits into the host cell cytosol than the binary actin ADP-ribosylating toxins.

So far, the molecular mode of action of EGA, which is responsible for the protective effects of this compound against bacterial protein toxins, is not known. Here, we discovered that EGA inhibits the pH-dependent membrane transport of C2I, the enzyme component of C2 toxin. However, it is not clear whether this mode of action is the reason for the inhibitory effects of EGA against the other toxins, as well.

From the present experiments and the previous finding of the low toxicity in mice (64), EGA emerges as an attractive lead compound to develop novel broad-spectrum inhibitors against a variety of bacterial protein toxins, including the binary actin ADP-ribosylating toxins from clostridia, because it efficiently protects cells without obvious cytotoxic effects.
